# Ungulate browsers promote herbaceous layer diversity in logged temperate forests

**DOI:** 10.1002/ece3.2223

**Published:** 2016-06-12

**Authors:** Edward K. Faison, Stephen DeStefano, David R. Foster, Glenn Motzkin, Joshua M. Rapp

**Affiliations:** ^1^ HighsteadPO Box 1097ReddingConnecticut06875; ^2^ U.S. Geological SurveyMassachusetts Cooperative Fish & Wildlife ResearchUniversity of Massachusetts Amherst160 Holdsworth WayAmherstMassachusetts01003; ^3^ Harvard ForestHarvard University324 North Main StreetPetershamMassachusetts01366; ^4^ The Conway School332 South Deerfield RoadConwayMassachusetts01341

**Keywords:** Browsing, disturbance, herbivory, moose, species richness, white‐tailed deer

## Abstract

Ungulates are leading drivers of plant communities worldwide, with impacts linked to animal density, disturbance and vegetation structure, and site productivity. Many ecosystems have more than one ungulate species; however, few studies have specifically examined the combined effects of two or more species on plant communities. We examined the extent to which two ungulate browsers (moose [*Alces americanus*]) and white‐tailed deer [*Odocoileus virginianus*]) have additive (compounding) or compensatory (opposing) effects on herbaceous layer composition and diversity, 5–6 years after timber harvest in Massachusetts, USA. We established three combinations of ungulates using two types of fenced exclosures – none (full exclosure), deer (partial exclosure), and deer + moose (control) in six replicated blocks. Species composition diverged among browser treatments, and changes were generally additive. Plant assemblages characteristic of closed canopy forests were less abundant and assemblages characteristic of open/disturbed habitats were more abundant in deer + moose plots compared with ungulate excluded areas. Browsing by deer + moose resulted in greater herbaceous species richness at the plot scale (169 m^2^) and greater woody species richness at the subplot scale (1 m^2^) than ungulate exclusion and deer alone. Browsing by deer + moose resulted in strong changes to the composition, structure, and diversity of forest herbaceous layers, relative to areas free of ungulates and areas browed by white‐tailed deer alone. Our results provide evidence that moderate browsing in forest openings can promote both herbaceous and woody plant diversity. These results are consistent with the classic grazing‐species richness curve, but have rarely been documented in forests.

## Introduction

Large herbivores are leading drivers of terrestrial plant composition and dynamics and therefore important determinants of biodiversity and a host of ecosystem services (Diaz et al. 2007; Hegland et al. 2013; Borer et al. 2014). In addition to site conditions (e.g., disturbance, vegetation structure, and soil productivity), herbivore density is an important determinant of ungulate impacts on plant communities. The classic grazing curve suggests that intermediate levels of herbivory should result in the highest species richness (intermediate disturbance hypothesis), with richness lowest at the two grazing extremes (Grime 1973). Although the humped‐back grazing curve has been documented in grassland ecosystems (Mwendera et al. 1997; Olff and Ritchie 1998; Suominen et al. 2003), it has rarely been reported in forests, in part because (1) forest ungulates are generally not stocked at extremely high levels (Hegland et al. 2013), (2) few experiments have manipulated multiple levels of ungulate densities in forests (Wisdom et al. 2006), and (3) because ungulate‐forest experiments have predominantly occurred in areas of moderate‐to‐high deer densities (>8.5 km^−2^; Russell et al. 2001).

Although many studies have examined forest ecosystems with more than one ungulate species (e.g., Ammer 1996; Gill and Morgan 2010; Kuijper et al. 2010), few studies have specifically examined the effects of different numbers of ungulate species on forest and other plant communities (Ritchie and Olff 1999; Hester et al. 2006). The effects of two or more ungulates on plant composition may be additive or compensatory, depending on the species’ diets (Ritchie and Olff 1999; Hester et al. 2006). Additive effects occur when multiple herbivores consume primarily the same plant species and therefore alter the community in the same direction as a single herbivore (Ritchie and Olff 1999). Compensatory effects occur if two herbivore species consume primarily different plant species so that their individual effects on species’ relative abundance result in little net effect on composition (Ritchie and Olff 1999).

Studies on the effects of two or more herbivores on forest dynamics are particularly relevant today because of recent range expansions and recolonization of previously extirpated forest ungulates (Foster et al. 2002; Gill 2006; Kuijper et al. 2010). In the late 20th century, moose recolonized much of their former southern range in the northeastern deciduous forest region (Faison et al. 2010; Wattles and DeStefano 2011). Almost nothing is known about how moose interact with white‐tailed deer to shape the structure and composition of eastern temperate forests. Much of the cool northeastern section of the eastern deciduous forest where moose occur is characterized by low‐to‐moderate densities (i.e., 3–7 km^−2^) of white‐tailed deer. Deer at these densities typically have only minor effects on forest regeneration (Tilghman 1989; Healy 1997); but can promote or reduce plant species diversity depending on site disturbance and plant life form (Royo et al. 2010). Moose, at their southern range limit in northeastern North America, occur at very low densities (~0.2 km^−2^), and it is unclear to what extent, if any, moose have shifted plant communities in ways that are different from deer alone. Differences in diet could contribute to a shift. Although both animals are generalist browsers with considerable overlap in diet, moose are almost exclusively (90%) browsers, whereas deer diets are more evenly comprised of browse (60%) and herbaceous material (40%; Renecker and Schwartz 1997). Habitat could also be an important factor. Moose are particularly associated with disturbed forest openings (i.e., burned or logged areas) in which to forage (Peek 1997; Geist 1998), whereas deer tend to browse more evenly across disturbed and undisturbed areas (Degraaf and Yamasaki 2001). Finally, the great size (on average, 7 times greater in mass than white‐tailed deer; Jones et al. 2009) and browse consumption of moose combined with their preference for disturbed areas could result in much higher browsing intensities in forest openings than might be expected from their ambient densities (Persson et al. 2005; Faison et al. 2016). Thus, it seems reasonable to predict that the arrival of the larger herbivore to areas formerly browsed only by deer should result in important differences to the plant community.

Here, we examine the effects of three combinations of large browsers (zero, deer, and deer + moose) on the composition, diversity, and structure of temperate forest herbaceous layers 5–6 years after canopy removal from logging in central New England, USA. We predicted that the addition of moose would generally influence the vegetation in the same direction as deer alone (additive effects), despite some differences in the animals’ diet (Renecker and Schwartz 1997), but to a greater extent. In turn, we predicted that browsing by deer + moose would increase herbaceous species richness, while at the same time reducing woody plant richness (Royo et al. 2010; Hegland et al. 2013).

## Materials and Methods

### Study area and species

The physiography of central Massachusetts is characterized by rolling plateaus with hills, and the climate is humid with warm summers and cold winters (Brouillet and Whetstone 1993; US EPA 2015). Mean annual precipitation ranges from 97 to 127 cm per year, and mean temperature ranges from −12°C to −0.5°C in January and 14°C to 28°C in July. Mature forest vegetation is characterized by transition hardwood forests – mixed oak (*Quercus* spp.), red maple (*Acer rubrum*), black birch (*Betula lenta*), and beech (*Fagus grandifolia*) – with significant components of eastern hemlock (*Tsuga canadensis*) and white pine (*Pinus strobus*; Foster et al. 2004; USDA 2015). Timber harvesting, exotic forest insects, and pathogens including hemlock woolly adelgid (*Adelges tsugae*), beech bark disease (*Cryptococcus fagisuga* and *Nectria* spp.), chestnut blight (*Cryphonectria parasitica*), and gypsy moths (*Lymantria dispar*); and meteorological events (ice and windstorms) are the prevalent disturbances in the region (Foster et al. 2004). White‐tailed deer densities are estimated at 3.9–5.8 km^−2^ for north central Massachusetts (McDonald et al. 2007; David Stainbrook, Massachusetts Division of Fish and Wildlife *pers. comm*.), and moose densities are estimated to be about 0.2 km^−2^.

### Experimental design

**Figure 1:**
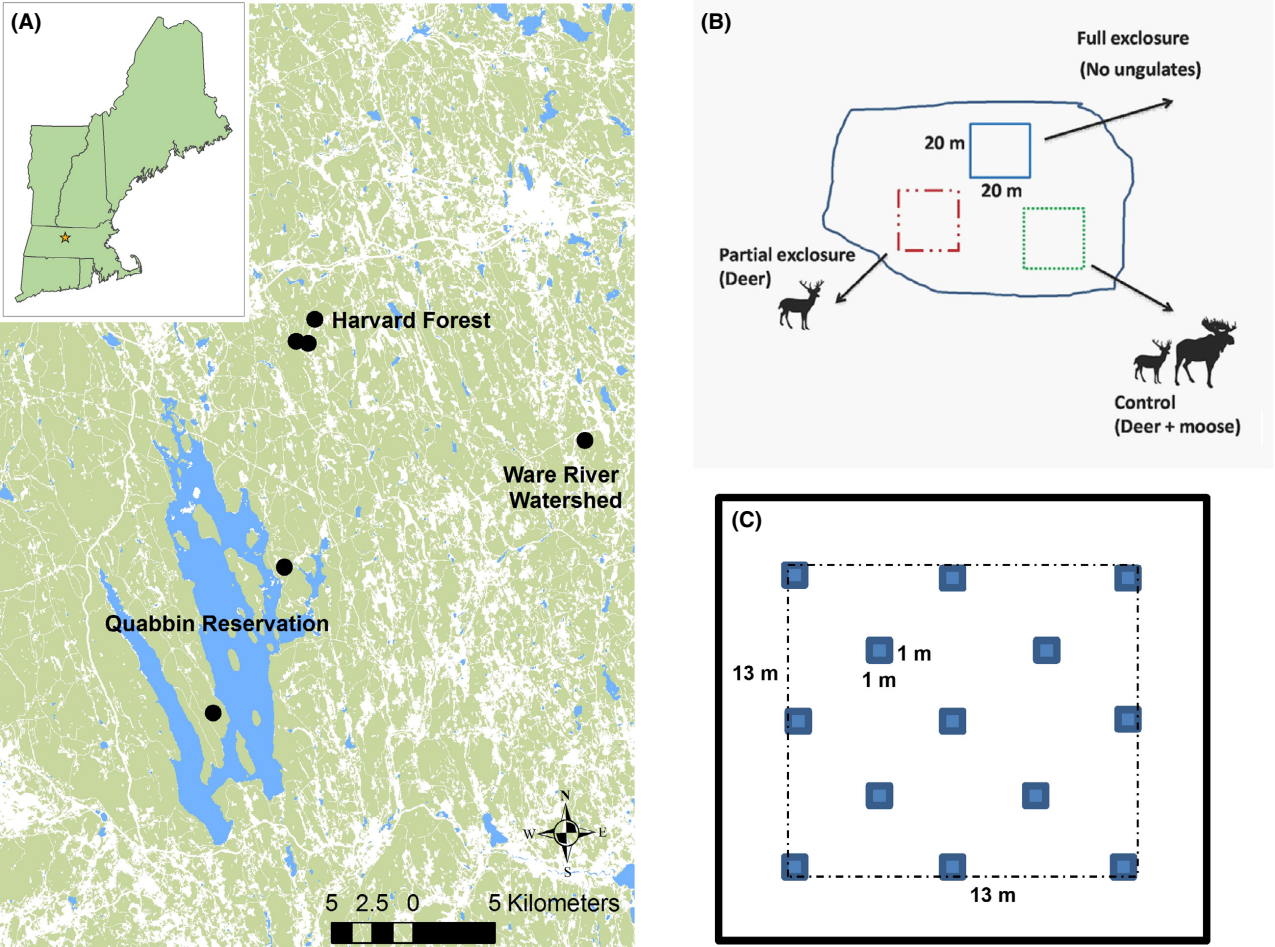
Map showing (A) location of study area and six study blocks in New England, USA; (B) experimental design showing three browser treatments; and (C) layout of sampling quadrats and larger 13 × 13 m sampling area within each treatment plot.

In 2007–2008, six mixed conifer‐hardwood stands that had been clear‐cut within the past 3–6 months at the Harvard Forest and the Quabbin and Ware River Watershed forests in Central Massachusetts were selected as study sites (Fig. 1A). Four of the sites were former conifer plantations (larch [*Larix* spp.], red pine [*Pinus resinosa*] or spruce [*Picea* spp.]) with mixed native hardwoods, and the other two were red oak (*Quercus rubra*)–white pine‐dominated stands with red maple in the understory (Table 1). In each site, a randomized block with three treatment levels of large herbivores – no‐ungulates (full exclosure), deer (partial exclosure), and deer + moose (control) was established (Fig. 1B). A fourth treatment that excluded deer but was open to the larger moose was not feasible (cf. Ritchie and Olff 1999). Blocks were at least 700 m apart from one another. The 2.5‐m‐tall exclosures were made of high‐tension wire game fence with 15‐cm grid mesh. The full exclosure was fenced to the ground; the partial exclosure had a 60‐cm opening between the bottom of the fence and the ground surface that excluded moose but allowed access to deer and all other wildlife; and the control plot was unfenced and open to both browsers (Fig. 1B). The 15‐cm wire mesh of the fence enabled access to small mammals including lagomorphs and rodents.

**Table 1:** Characteristics of study site blocks in Central Massachusetts, USA

Block	Location	Previous overstory composition	Exclosures built	Age of plot at time of sampling (years)
Dana	Quabbin Reservation Forest	Oak–red maple–black birch	November 2007	5.6
Fisher	Harvard Forest	Red pine–white pine, black birch–red maple	July 2008	4.9
Locust	Harvard Forest	Red pine–red maple–red oak	June 2008	5.1
Prescott	Quabbin Reservation Forest	Oak–red maple–black birch	November 2007	5.7
Prospect	Harvard Forest	Spruce–black cherry–red maple	October 2008	4.8
Ware	Ware River Reservation Forest	Pine–larch–hardwoods	December 2007	5.6

### Vegetation sampling

At the start of the experiment, no woody stems above 1.5 m in height occurred in any of the plots, and virtually all remaining woody stems were less than 1 m. Exclosure and control plots were 20 × 20 m in size and separated by 10–90 m. In 2013, 5–6 years after treatment, we established 13, 1 m^2^ subplots in a systematic grid in the center of each plot. Five rows were established with 3 subplots on the outer and middle rows and two subplots in the second and fourth rows. Subplots were positioned 6 m apart within the same row and 4.5 m apart between rows and at least 4 m from the edge of the fence (Fig. 1C). At each 1 × 1 m subplot, all vascular plants in the forest floor layer were recorded by a single observer (original field data collected by Glenn Motzkin archived at Harvard Forest). We defined “herbaceous layer” as all plants (herbs, shrubs, and trees) <2 m in height (Oliver and Larson 1996; Carson et al. 2014). Percent cover was estimated for each species and for each plant group (i.e., woody plants, graminoids, and forbs) in one of 7 cover classes (1 = <1%, 2 = 1–5%, 3 = 6–15%; 4 = 16–25%, 5 = 26–50; 6 = 51–75%, 7 = 76–100%). Because many of the woody stems had recruited above 2 m by the time of sampling, we also estimated the percent cover of woody plants ≥2 m in height. In addition, we performed a 20‐min “meander” survey throughout the central 13 × 13 m of each plot and recorded the presence of all woody and herbaceous plant species that did not occur in the subplots (Fig. 1C; Huebner 2007; Goetsch et al. 2011). All data were collected in June and July of 2013, and treatments within a block were sampled in the same week. Nomenclature followed Cullina et al. (2011).

### Ungulate activity

To test the integrity of the experimental design for the presence of the different ungulate species, remote cameras (Reconyx, Inc. [Holmen, Wisconsin] and Cuddeback, Inc. [Greenbay, Wisconsin]) were mounted inside each partial exclosure and toward each control plot between 2008 and 2011. Cameras were discontinued after 2011 because the vegetation had grown to a height that effectively blocked the camera's ability to detect animals. Ungulate pellet groups were counted in 2012 in 25, 4 m^2^ subplots in each plot. Pellet groups were defined as having at least 15 individual pellets to avoid over‐counts from scattered individual pellets (Eschtruth and Battles 2008).

### Data analysis

We focused our analysis on native species because exotic species occurred in only a small subset of the subplots (17 of 234) and were unimportant in the total flora of our study plots. We used percent cover as a measure of vegetation abundance. We first converted cover classes to percent cover midpoints, and then calculated the mean percent cover for species and growth form groups (i.e., ferns, forbs, graminoids, woody plants) across the 13, 1 × 1 m subplots in each plot. To test for significant differences in community composition among treatments, we used permutation multivariate analysis of variance using distance matrices (Bray) with the adonis function (package vegan, Oksanen et al. 2015), grouped by block (1000 permutations). Species abundances (% cover) were entered into the multivariate test (Motzkin et al. 1999). Rare species that occurred in only 1 of the 18 treatment plots (5.5%) were removed prior to analysis (McCune et al. 2002). To further examine the potential effects of ungulate browsing on species composition from a successional context, we calculated the combined abundance of herb and shrub species characteristic of forest habitats in each plot and the combined abundance of herb and shrub species characteristic of open/disturbed habitats. We used habitat descriptions from the New England Wild Flower Society (2015) and Haines (2011) to categorize species into different habitat/successional groups (Table 2). *Forest indicator species* were (1) required to be listed as occurring in forests and (2) could not be listed as occurring in anthropogenic or disturbed habitats, marshes, or meadows and fields. *Open/disturbed habitat species* (1) could not be listed as occurring in forests and (2) were required to be listed as occurring in anthropogenic or disturbed habitats, marshes, or meadows and fields (Table 2).

**Table 2:** Herb and shrub species associated with forest and open/disturbed habitats used in species composition analysis

Forest herbs	Forest shrubs	Open/disturbed herbs	Open/disturbed shrubs
*Aralia nudicaulis*	*Corylus cornuta*	*Aralia hispida*	*Comptonia peregrina*
*Carex swanii*	*Sambucus racemosa*	*Carex vestita*	*Prunus virginiana*
*Dryopteris intermedia*	*Swida alternifolia*	*Carex normalis*	*Rhus hirta*
*Lysimachia borealis*	*Vaccinium corymbosum*	*Carex lurida*	*Rubus allegheniensis*
*Medeola virginiana*	*Viburnum nudum*	*Carex scoparia*	*Rubus flagellaris*
*Mitchella repens*		*Danthonia compressa*	*Rubus hispidus*
*Parathelypteris noveboracensis*		*Fragaria virginiana*	
*Uvularia sessilifolia*		*Juncus effusus*	
		*Lysimachia quadrifolia*	
		*Potentilla simplex*	
		*Solidago rugosa*	

Species selected from 51 common species that occurred in at least two treatment plots. Habitat associations determined from Haines (2011) and New England Wild Flower Society (https://gobotany.newenglandwild.org/).

We examined species richness for herbs and woody plants at two scales in each plot: subplot scale (mean number of species in 13, 1 m^2^ quadrats) and plot scale (number of species 169 m^‐2^). Although these measurements are technically species density (Gotelli and Colwell 2001), we hereafter refer to species density as “species richness” for the sake of clarity. We used linear mixed effects models (package lme4; Bates et al. 2014) in R version 2.15.2 (R Core Team 2014) with ungulate treatment as fixed effect and block as random effect to examine species richness, abundance of forest and open indicator species, and abundance of different growth form groups to three levels of browsers. We also examined models including variation in herbivore abundances across the blocks (using pellet count indices) as a covariate, but in no instance did these models fit the data better than the basic treatment model when compared by AIC. We used either normal or log normal models for each response variable after examining the residuals to determine the best fit. For hypothesis tests of treatment effects, we used likelihood ratio tests (LRTs). For significant results (*P* < 0.05) of treatment, we performed pairwise comparisons between the three treatment pairs by simulating the posterior distribution 10,000 times to calculate 95% confidence intervals and approximate P‐values for the fixed effects (Gelman and Hill 2007; Bagchi et al. 2011; Rapp et al. 2013). This test further validated the results obtained from the LRT. Alpha was set = 0.05.

## Results

Remote cameras detected deer in every partial exclosure and control plot and moose in every control plot but none of the partial exclosure plots. Bobcat (*Lynx rufus*), black bear (*Ursus americanus*), coyote (*Canis latrans*), and wild turkey (*Meleagris gallopavo*) were all observed inside the partial exclosures, as well as in the control areas, suggesting the partial exclosure plots were permeable to all animal species except for moose. Deer pellet groups were detected in all of the partial exclosure and control plots, and moose pellets were only detected in the control plots.

### Effect on species composition and structure

**Figure 2:**
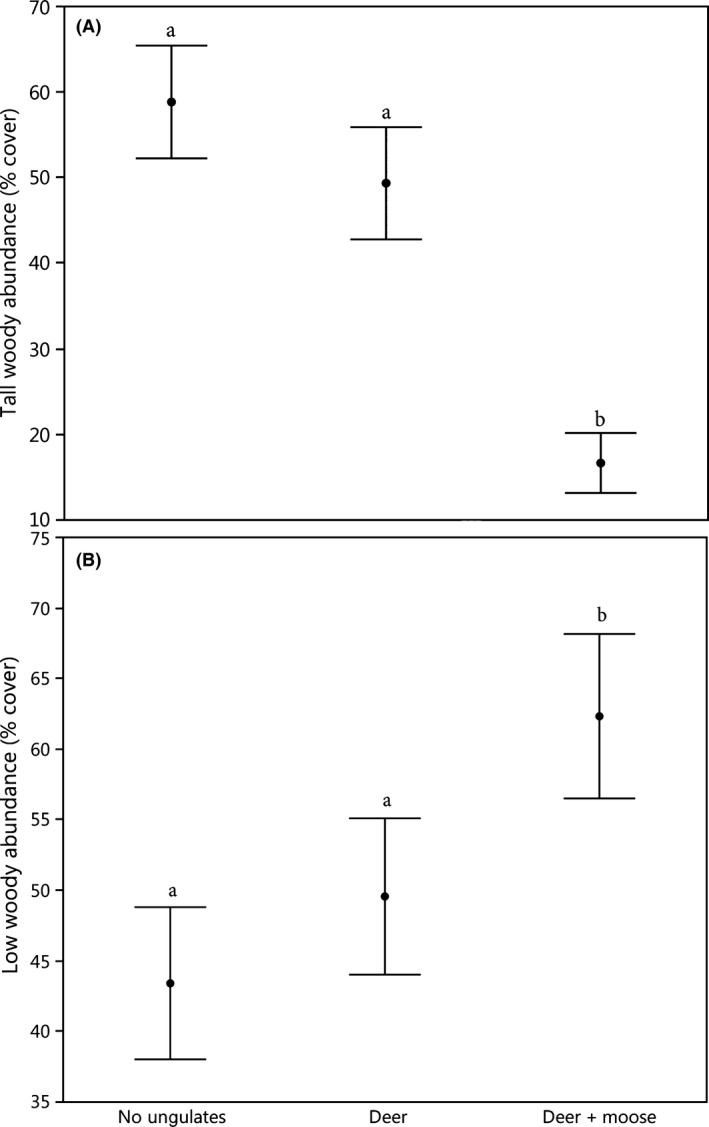
Effect of browsers on the abundance of woody plants (A) above the herbaceous layer (≥ 2 m in height; LRT *χ* ^2^ = 23.2; df = 2; *P* < 0.0001) and (B) within the herbaceous layer (<2 m in height; LRT *χ* ^2^ = 13.78; df = 2; *P* = 0.001). Treatment means with the same letter do not differ significantly. Bars represent mean ± SE.

Browsing by deer + moose significantly reduced woody plant abundance above 2 m in height (*P* < 0.0001; Fig. 2A) and simultaneously increased woody plant cover below 2 m in height relative to deer browsing alone and ungulate exclusion (*P* = 0.001; Fig. 2B). Across the 18 plots in 6 blocks, 124 native vascular plant taxa below 2 m in height were recorded to species or genus, including 8 ferns, 31 forbs, 25 graminoids, and 60 woody plants (tree, shrubs, and lianas). Sixty‐one species of trees, shrubs, and herbs were common, occurring in at least two of the 18 plots. Analysis of ecological distance revealed a significant difference in species composition among treatments (Adonis *F* = 0.78; *R* ^2^ = 0.09; *P* = 0.004).

**Figure 3:**
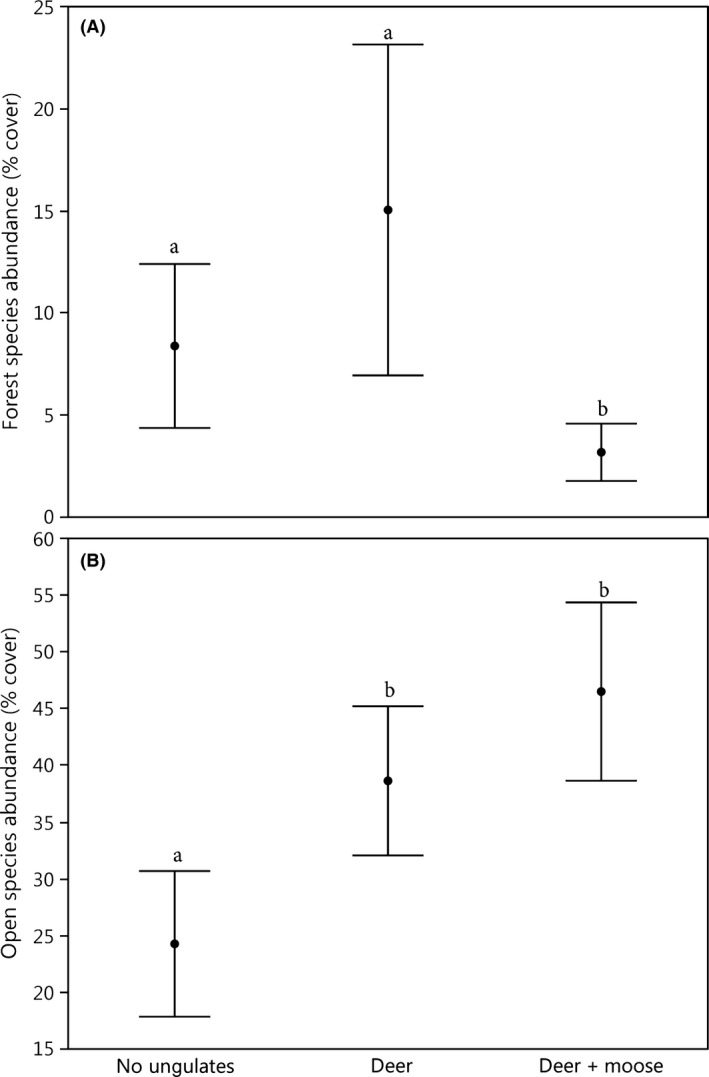
Effect of browsers on the combined abundance of (A) 13 herb and shrub species affiliated with undisturbed/forest habitat (LRT = *χ* ^2^ = 9.81; df = 2; *P* = 0.007) and (B) 18 herb and shrub species associated with disturbed/open habitats (LRT *χ* ^2^ = 9.20; df = 2; *P* = 0.010; *P* = 0.01). Treatment means with the same letter do not differ significantly. See Table 2 for list of indicator species. Bars represent mean ± SE.

Forb abundance and graminoid abundance did not differ among treatments (Table 3), but fern abundance (mostly eastern hay‐scented fern [*Dennstaedtia punctilobula*]; 50% and evergreen wood fern [*Dryopteris intermedia*]; 44%) was higher in deer plots and no‐ungulate plots than in deer + moose plots (LRT *χ* ^2^ = 7.1; df = 2; *P* = 0.028; Table 3). Deer and no‐ungulate plots did not differ in fern cover. Total *Rubus* abundance was greater in deer + moose plots than in no‐ungulate plots (LRT *χ* ^2^ = 8.98; df = 2; *P* = 0.01; Table 3). Other treatment combinations did not differ significantly. Deer + moose plots supported less than half the abundance of forest herb and shrub indicator species, on average, than did no‐ungulate and deer plots (LRT = *χ* ^2^ = 9.81; df = 2; *P* = 0.007; Fig. 3A). In contrast, abundance of herb and shrub species characteristic of open/disturbed habitats was almost twice as high in deer + moose plots compared to no‐ungulate plots (LRT *χ* ^2^ = 9.20; df = 2; *P* = 0.010; Fig. 3B). Open/disturbed indicator species were also significantly more abundant in deer than in no‐ungulate plots (Fig. 3B).

**Table 3:** Mean abundance (% cover) of common plant species and growth form groups in the herbaceous layer (<2 m in height) by ungulate browser treatment

Species	No‐ungulates	Deer	Deer + moose
Woody plants^a^	43.4 (5.4)^a^	49.6 (5.5)^a^	62.3 (5.8)^b^
*Acer rubrum*	3.50 (0.42)	4.0 (1.35)	5.43 (1.10)
*Amelanchier spp*.	0.17 (0.14)	0.27 (0.17)	0.14 (0.13)
*Betula lenta*	0.29 (0.18)	1.40 (0.70)	3.0 (1.67)
*Betula papyrifera*	0.31 (0.21)	0.17 (0.17)	0.49 (0.32)
*Comptonia peregrina*	0.26 (0.29)	0.63 (0.49)	2.55 (1.61)
*Fraxinus americana*	0.13 (0.15)	0	0.99 (0.59)
*Gaylussacia baccata*	1.36 (1.44)	0.66 (0.66)	2.16 (1.62)
*Ilex verticillata*	0.17	0	0.60 (0.38)
*Pinus strobus*	0.88 (0.62)	0.15 (0.13)	1.00 (0.83)
*Prunus pensylvanica*	0.59 (0.55)	1.14 (0.42)	0.81 (0.39)
*Prunus serotina*	0.82 (0.41)	0.54 (0.31)	1.88 (1.0)
*Quercus rubra*	0.97 (0.41)	2.18 (1.64)	1.20 (0.57)
*Quercus velutina*	1.05 (0.54)	0.17 (0.13)	0.04 (0.04)
*Rubus allegheniensis*	10.51 (6.32)	17.31 (4.39)	23.06 (8.7)
*Rubus flagellaris*	0	0.66 (0.52)	3.91 (3.86)
*Rubus hispidus*	10.82 (7.0)	17.47 (10.11)	12.31 (8.16)
*Rubus idaeus*	7.07 (4.0)	4.22 (1.68)	7.28 (2.66)
*Rubus total* a	28.41 (5.02)^a^	39.66 (6.64)^ab^	46.55 (6.73)^b^
*Vaccinium angustifolium*	3.70 (2.17)	2.28 (1.46)	2.1 (1.73)
*Vaccinium carymbosum*	1.15 (0.59)	0.97 (0.62)	0.30 (0.26)
*Vaccinium pallidum*	1.49 (1.58)	0.02 (0.02)	1.99 (1.83)
*Vitis labrusca*	0.17 (0.14)	0.01 (0.01)	0.01 (0.01)
Forbs	14.93 (0.74)	18.67 (3.81)	13.21 (2.97)
*Aralia hispida*	0.01 (0.01)	0.74 (0.74)	0.40 (0.35)
*Aralia nudicaulis*	1.36 (1.01)	5.0 (2.89)	0.59 (0.54)
*Lysimachia borealis*	0.83 (0.70)	0.68 (0.38)	0.45 (0.20)
*Lysimachia quadrifolia*	0.43 (0.27)	1.12 (0.70)	0.95 (0.66)
*Maianthemum canadense*	5.30 (0.93)	3.66 (0.79)	3.24 (2.17)
*Medeola virginiana*	0.04 (0.04)	0.01 (0.01)	0.05 (0.04)
*Mitchella repens*	0.13 (0.09)	0.04 (0.04)	0.04 (0.03)
*Potentilla simplex*	0.15 (0.17)	0.33 (0.29)	1.41 (1.05)
*Rumex acetosella*	0.01 (0.01)	0.06 (0.04)	0.01 (0.01)
*Solidago rugosa*	0.26 (0.29)	0.14 (0.13)	0.19 (0.19)
*Uvularia sessilifolia*	0.23 (0.15)	0.16 (0.14)	0.02 (0.02)
Graminoids	4.9 (2.01)	2.56 (0.56)	7.59 (3.0)
*Carex debilis*	0.08 (0.06)	0.23 (0.17)	0.64 (0.60)
*Carex pensylvanica*	2.35 (2.42)	1.0 (0.33)	5.36 (3.02)
*Carex scoparia*	0.32 (0.35)	0.01 (0.01)	0.46 (0.33)
*Carex swanii*	0.28 (0.28)	0.31 (0.31)	0.47 (0.31)
*Carex vestita*	1.43 (1.04)	0.03 (0.03)	1.15 (1.15)
*Danthonia compressa*	0.01 (0.01)	0.04 (0.04)	0.06 (0.05)
Ferns^a^	7.0 (1.96)^a^	9.19 (4.31)^a^	5.27 (3.35)^b^
*Dennstaedtia punctilobula*	5.55 (2.75)	6.78 (4.65)	4.37 (3.38)
*Dryopteris intermedia*	1.39 (0.61)	2.28 (1.36)	0.74 (0.55)

Only species that occurred in at least 4 of the 18 treatment plots were included. Standard errors are in parentheses. Total growth form groups were compared among treatments using Likelihood Ratio Tests.

a *P* < 0.05.

Treatment means with different superscript letters are significantly different.

### Species richness: subplot scale (1 m^2^)

**Figure 4:**
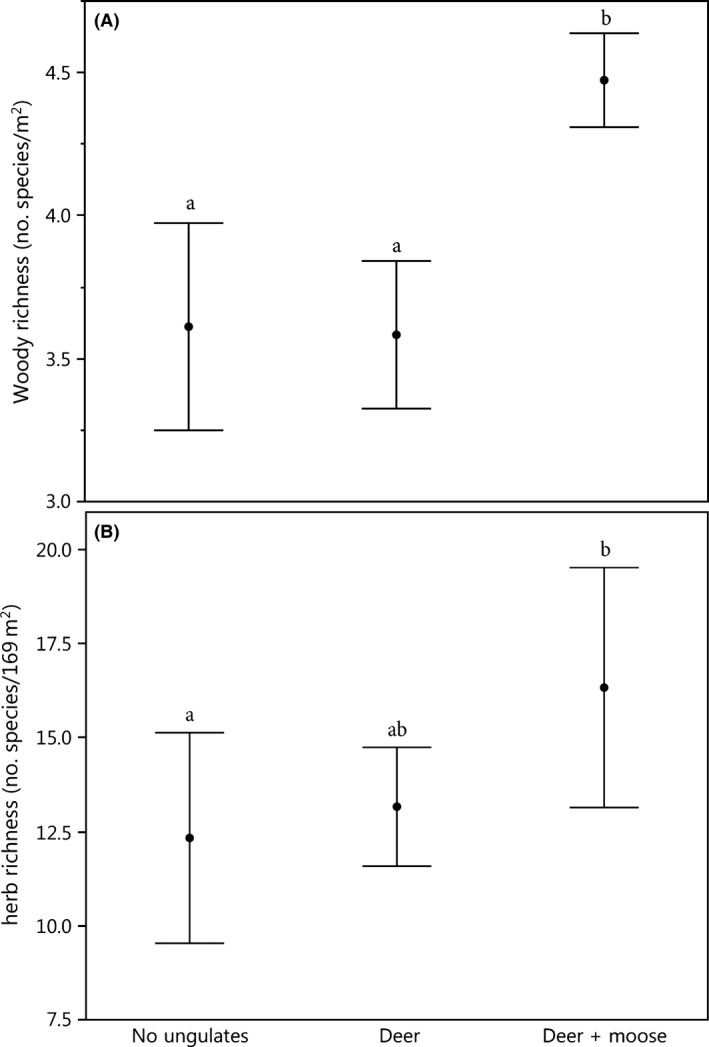
Effect of browsers on (A) native woody plant richness at the subplot scale (mean no. of species of trees, shrubs, and lianas in 13, 1 m^2^ quadrats; (LRT *χ* ^2^ = 10.21; df = 2; *P* = 0.006); and (B) native herbaceous species richness at the plot scale (no. species of forbs, graminoids, and ferns 169 m^−2^; LRT = *χ* ^2^ = 8.6; df = 2; *P* = 0.013). Treatment means with the same letter do not differ significantly. Bars represent mean ± SE.

Deer + moose significantly increased woody species richness relative to areas with just deer or no‐ungulates (*P* = 0.006; Fig. 4A). The presence of deer alone had little effect on woody richness relative to areas without ungulates. There was no significant difference in total native species among no‐ungulate (7.1 species m^−2^; SE = 0.70), deer (7.3 species m^−2^; SE = 0.4) and deer + moose plots (8.2 species m^−2^; SE = 0.48; LRT *χ* ^2^ = 4.59; df = 2; *P* = 0.10). Native herbaceous and forb density were similar among treatment plots.

### Species richness: plot scale (169 m^2^)

Native herbaceous plant richness (combined number of forbs, ferns, and graminoid species) was greater by 3 and 4 species, on average, in deer + moose plots than in deer and no‐ungulate plots, respectively (*P* = 0.013; Fig. 4B). Graminoid species richness was higher in deer + moose plots (7.5 species; SE = 1.8) and deer plots (5.2 species; SE = 0.79) than in no‐ungulate plots (4.5 species; SE = 1.8; LRT *χ* ^2^ = 11.99; df = 2; *P* < 0.002). Neither forb nor woody species richness differed among treatments.

## Discussion

Two ungulate browsers had strong additive effects on the structure and composition of temperate forest herbaceous layers 5–6 years after complete canopy removal from logging. To our knowledge, this experiment is the first to examine the individual effects of one ungulate browser vs the combined effects of two browsers on herbaceous layer vegetation in temperate forests of North America and perhaps temperate forests anywhere. Because our study design only enabled us to separate out the effects of deer, and not moose, we did not attempt to examine individual species effects on the vegetation nor to tease apart herbivore composition from herbivore density (cf. Ritchie and Olff 1999; Manier and Hobbs 2007; Lagendijk et al. 2011). Rather, our goals were to examine how the recolonization of low densities of moose to a system browsed by low densities of deer altered the plant community, and how moderate intensity browsing by deer + moose altered the community differently from low intensity browsing by deer alone.

Consistent with our predictions, browsing by deer+ moose generally altered species composition, abundance, and diversity in the same direction – but to a much greater extent – than deer alone. Our most notable results were an increase in herbaceous (plot scale) and woody (subplot scale) plant richness by the addition of moose, which contrasted sharply with the relatively little effect by deer browsing alone. These results appeared to provide support for the classic grazing curve – in which species richness increases from low to moderate grazing/browsing pressures (Grime 1973) – previously documented only sparingly in temperate forest ecosystems browsed by wild ungulates (e.g., Schreiner et al. 1996; Suzuki et al. 2008; Royo et al. 2010).

The increase in herb richness by deer + moose, presumably as a result of the browsers depleting the woody canopy and reducing competition for shorter statured herbs (Hester et al. 2006; Royo et al. 2010), shows parallels to ungulates promoting herb diversity in recently burned grasslands by reducing dominant grasses (Collins et al. 1998; Knapp et al. 1999). However, in contrast to burned and grazed grasslands in which forbs are the major driver of diversity, graminoids were the major driver of herb richness in our forest plots. Graminoids are preferred by ungulate grazers over forbs, whereas the reverse is generally true for ungulate browsers (Renecker and Schwartz 1997). A number of authors have noted increases in graminoid abundance with increased ungulate browsing in forests (e.g., Kirby 2001; Rooney 2009).

Interestingly, several studies that documented increased richness of herbs with browsing in forests show simultaneous declines in woody/shrub richness (Schreiner et al. 1996; Royo et al. 2010; Hegland et al. 2013). Our results, however, showed an increase in both herb and low woody plant richness with greater herbivory by deer + moose. A positive effect by browsing on woody plant diversity is uncommon in forests (but see Risenhoover and Maass 1987 and Pekin et al. 2014), as most studies have reported declines in woody richness in response to increased browsing (Horsley et al. 2003; Gill 2006; Nuttle et al. 2013). Kuijper et al. (2010) reported higher species diversity in the smallest size class of tree seedlings combined with lower diversity in the larger size class of seedlings with browsing. In a parallel manner, woody richness in our study area increased below 2 m in height, but generally declined in both deer and deer + moose treatments above 2 m in height (Faison 2015). In other words, heavy browsing by deer + moose reduced the height of many stems and species below 2 m that would otherwise have grown taller in the absence of browsing (cf. Risenhoover and Maass 1987; McInnes et al. 1992). With very high densities of browsers, a decline in woody richness appears to occur even in the lowest plant layers (Horsley et al. 2003; Nuttle et al. 2013); however, these studies focused only on tree species, rather than combined trees and shrubs, complicating comparison with ours.

Divergent herbaceous layer communities that developed among plots exposed to different assemblages of large herbivores reveal the important role that ungulates, even at low‐to‐moderate densities, play in altering forest composition and succession following disturbance (Royo et al. 2010). Areas browsed by ungulates (both deer alone and deer + moose) supported a greater abundance of herbs and low shrubs associated with open/disturbed habitats than did plots protected from herbivory. At the same time, herb and shrub assemblages of undisturbed forest habitats were more than twice as abundant in no‐ungulate and deer plots than in deer + moose plots. Hence, browsers maintained vegetation composition in an earlier successional state than in plots protected from browsing (cf. Risenhoover and Maass 1987; Bergquist et al. 1999). Indeed, browsing by deer + moose is likely to prolong the stand initiation stage of forest succession in which shrubs and herbaceous plants proliferate following disturbance and prior to canopy closure (Oliver and Larson 1996). Dramatic reductions of tall woody plants above 2 m in height by browsers, resulting in greater light and a more open grown structure in control plots, was likely one mechanism driving divergent herb and low shrub communities (cf., Persson et al. 2000; Royo et al. 2010; Roberts and Gilliam 2014). Additionally, trampling by moose in control plots may have resulted in greater soil compaction and damage to some of the more sensitive forest herbs and shrubs (Persson et al. 2000; Hester et al. 2006; Heckel et al. 2010). Forest specialist forbs are generally of greater threat to local extinctions than many disturbance‐adapted generalists and are often preferred by deer (Wiegmann and Waller 2006). Hence, the extent to which forest herb and shrub specialists in browsed areas are able converge in abundance with those found in ungulate excluded plots will be important to track as these stands develop over time.

Three species of *Rubus* (*R. allegheniensis*,* R. hispidus*, and *R. flagellaris*) accounted for over 75% of open‐field species abundance. Increased Rubus abundance with greater browsing pressure contrasts with earlier studies of white‐tailed deer – *Rubus* – dynamics in logged eastern deciduous forests (e.g., Horsley et al. 2003; Royo et al. 2010) and of ungulate‐*Rubus* dynamics in European temperate forests (Kuiters and Slim 2002; Perrin et al. 2011) in which this shrub genus declined with increased browsing. One possible explanation for the opposing pattern in our study area is that moose, unlike deer, largely avoid *Rubus* in favor of other woody taxa (Telfer 1967; Belovsky 1981); therefore, this shrub is less likely to be reduced by moderate densities of deer + moose compared to moderate densities of deer alone (e.g., Royo et al. 2010). Deer at densities of ~4.5 km^−2^, as occurred in our study area, are generally too low to reduce *Rubus* spp. (cf. Tilghman 1989). Interestingly, Royo et al. (2010) attributed the increased herb richness in their study area, in part, to a browsing induced decline in the dominant *Rubus*. However, in our study area, herb richness increased along with an increase in *Rubus*, suggesting that this shrub was compatible with a diverse herb layer.

Greater cover of *Rubus* in deer + moose plots may, in turn, have contributed to the unexpectedly lower fern abundance in these same plots. Eastern hay‐scented fern (*Dennstaedtia punctilobula*), a codominant species in our study area, tends to increase with deer browsing in clearings, but to decline with greater abundance of *Rubus* spp. (Horsley and Marquis 1983). The other codominant fern species, evergreen wood fern (*Dryopteris intermedia*), is associated with undisturbed primary forests (Flinn 2014), and thus would be expected to grow less well in the relatively open grown structure of the control plots. Additionally, evergreen wood fern has been reported to decline in heavily browsed intact forests (Webb et al. 1956; Frerker et al. 2013), suggesting some sensitivity of this species to ungulate herbivory or trampling.

Increased browsing by deer + moose resulted in strong changes to the composition, structure, and diversity of forest herbaceous layers, relative to areas free of ungulates and areas browed by white‐tailed deer alone. Our results provide additional evidence that moderate browsing in forest openings often promotes plant diversity by the reduction of dominant woody plants. These results are consistent with the classic grazing‐species richness curve that has long been documented in grasslands, savannahs, and shrublands; but has rarely been documented in forests (Grime 1973; Hegland et al. 2013).

## Conflict of Interest

None declared.
